# Evidence-based guidelines for managing patients with primary ER+ HER2− breast cancer deferred from surgery due to the COVID-19 pandemic

**DOI:** 10.1038/s41523-020-0168-9

**Published:** 2020-06-08

**Authors:** Mitch Dowsett, Matthew J. Ellis, J. Michael Dixon, Oleg Gluz, John Robertson, Ronald Kates, Vera J. Suman, Arran K. Turnbull, Ulrike Nitz, Matthias Christgen, Hans Kreipe, Sherko Kuemmel, Judith M. Bliss, Peter Barry, Stephen R. Johnston, Samuel A. Jacobs, Cynthia X. Ma, Ian E. Smith, Nadia Harbeck

**Affiliations:** 1 Ralph Lauren Centre for Breast Cancer Research, Royal Marsden Hospital, London, UK; 2 Breast Cancer Now Toby Robins Research Centre, Institute of Cancer Research, London, UK; 3 Lester and Sue Smith Breast Center and Dan L. Duncan Comprehensive Cancer Center, Baylor College of Medicine, Houston, TX USA; 4 Department of Molecular and Cellular Biology, Baylor College of Medicine, Houston, TX USA; 5 Department of Medicine, Baylor College of Medicine, Houston, TX USA; 6 Edinburgh Breast Unit, Western General Hospital, Edinburgh, UK; 7 Bethesda Hospital, Breast Center Niederrhein, Mönchengladbach, Germany; 8 Westdeutsche Studiengruppe, Mönchengladbach, Germany; 9 Uniklinik Köln, Köln, Germany; 10 University of Nottingham, Royal Derby Hospital, Uttoxeter Road, Derby, UK; 11 West German Study Group, Mönchengladbach, Germany; 12 Division of Biomedical Statistics and Informatics, Department of Health Sciences Research, Mayo Clinic, Rochester, MN USA; 13 CRUK Edinburgh Centre, Institute of Genetics and Molecular Medicine, University of Edinburgh, Edinburgh, UK; 14 Medical School Hannover, Institute of Pathology, Hannover, Germany; 15 Kliniken Essen-Mitte, Essen, Germany; 16 Clinical Trials and Statistics Unit, The Institute of Cancer Research, London, UK; 17 Breast Unit, Royal Marsden Hospital, London, UK; 18 National Surgical Adjuvant Breast and Bowel Project Foundation, Pittsburgh, PA USA; 19 Division of Oncology, Department of Medicine, Washington University School of Medicine, St. Louis, MO 63110 USA; 20 Breast Center, Department of Obstetrics and Gynecology, and CCCLMU, LMU University Hospital, Munich, Germany

**Keywords:** Breast cancer, Breast cancer

## Abstract

Many patients with ER+ HER2− primary breast cancer are being deferred from surgery to neoadjuvant endocrine therapy (NeoET) during the COVID-19 pandemic. We have collated data from multiple international trials of presurgical endocrine therapy in order to provide guidance on the identification of patients who may have insufficiently endocrine-sensitive tumors and should be prioritised for early surgery or neoadjuvant chemotherapy rather than NeoET during or in the aftermath of the COVID-19 pandemic for safety or when surgical activity needs to be prioritized. For postmenopausal patients, our data provide strong support for the use of ER and PgR status at diagnosis for triaging of patients into three groups in which (taking into account clinical factors): (i) NeoET is likely to be inappropriate (Allred ER <6 or ER 6 and PgR <6) (ii) a biopsy for Ki67 analysis (on-treatment Ki67) could be considered after 2–4 weeks of NeoET (a: ER 7 or 8 and PgR <6 or b: ER 6 or 7 and PgR ≥6) or (iii) NeoET is an acceptable course of action (ER 8 and PgR ≥6). Cut-offs for percentage of cells positive are also given. For group (ii), a high early on-treatment level of Ki67 (>10%) indicates a higher priority for early surgery. Too few data were available for premenopausal patients to provide a similar treatment algorithm. These guidelines should be helpful for managing patients with early ER+ HER2− breast cancer during and in the aftermath of the COVID-19 crisis.

## Introduction

In many countries severely affected by the COVID-19 pandemic the surgical management of primary breast cancer is being confined to patients at highest risk for early disease progression. Thus, surgery for ER+ HER2− tumors (>70% of the overall breast cancer population) is being frequently deferred in favour of neoadjuvant endocrine therapy (NeoET) because of patient safety concerns and resource availability. In severely affected areas, it may be many months before surgery for patients on NeoET becomes available, raising concerns that this approach maybe suboptimal. A recent publication recommended approaches for the general management of breast cancer during the COVID-19 crisis and proposed the use of endocrine treatment and delay of surgery until after COVID-19 for T1N0ER+ HER2− breast cancer and considering this for T2 or N1 ER+ HER2− disease^1^.

NeoET has been used for many years to down-stage tumors for improved surgical outcome. Generally, NeoET provides good initial control of primary breast cancer for several months or even years, but local progression can occur, particularly with prolonged treatment and these events are associated with worse outcomes. Data from a meta-analysis of randomised clinical trials of immediate surgery with tamoxifen compared to tamoxifen alone (with surgery only in the event of local progression) for primary breast cancer in women aged 70 years or older alone shows a 29% (proportional) reduction in distant recurrence rates and a 27% (proportional) reduction in breast cancer mortality rates in those allocated surgery, without any increase in non-breast cancer deaths (EBCTCG personal communication). Thus, the accurate identification of patients most likely to be poorly controlled by NeoET would allow their surgery or systemic treatment to be prioritised during or in the aftermath of COVID19 according to local circumstances. Conversely, identification of those likely to experience adequate tumor control by NeoET over a period of surgical deferment would be reassuring.

In order to address these issues, we have formed a consortium of international study groups comprising some of the widest experience of NeoET globally. The immediate objective was to review the published data that underpins the application of NeoET in more normal circumstances and to collate this information with previously unpublished data from many thousands of patients in ongoing studies to produce a simple algorithm for patient management. Given the time sensitivity of this matter, the details of the populations and methods provided in this paper have been reduced compared with that we would normally provide. In due course, detailed data will be provided. Relevant details of the trials involved in one or more of our analyses, including the IRB approvals are given in Table 1.

**Table 1:** Relevant details of the clinical trials included.

Trial Name	NCT number	IRBs	NeoET		Number of patients	Reference
			duration	agents		
POETIC^a^	2338310	London-South East Research Ethics Committee	2 weeks	anastrozole or letrozole	339	5
PALLET	2296801	London-Fulham Research Ethics Committee	14 weeks	letrozole ± palbociclib	131	10
IL1839/223	N/A	Royal Marsden Hospital Ethics Committee	16 weeks	anastrozole ± gefitinib	176	9
ADAPT HR + /HER2-	1779206	University of Cologne Institutional Review Board	3 weeks	postmenopausal mainly AI	1925	11
				premenopausal mainly tamoxifen	1386	
Z1031	265759	NCI Central Institutional Review Board	16–18 weeks	anastrozole or exemestane or letrozole	214	7
ALTERNATE^b^	1953588	NCI Central Institutional Review Board	24 weeks	anastrozole or fulvestrant or combination	1299	12

^a^Patients included in case-control study from AI arm only.

^b^Patients included only for analysis of progression on NeoET.

We emphasise that the approach we propose is currently a temporary measure where clinical safety or resource issues related to the COVID19 pandemic impact the availability of surgery; it is not a substitute for standard surgical care once circumstances permit.

In creating these guidelines, we have been mindful of the following:

1. Interventions should fall within current clinical pathways, in terms of laboratory tests performed and patient care procedures as much as possible.
2. The patient selection process should be as straightforward for clinical staff to implement and relevant to as many patients as possible.
3. We should provide biomarker cut-off values for different scoring systems (eg Allred score, percentage cells positive) to make them internationally useable/applicable.
4. The guidance must be considered in conjunction with clinical factors (eg tumor burden, patient age and co-morbidities).

## Results and discussion

### Proposed triaging approach for remaining on NeoET, considering alternative treatments or additional diagnostic procedures

**Fig. 1:**
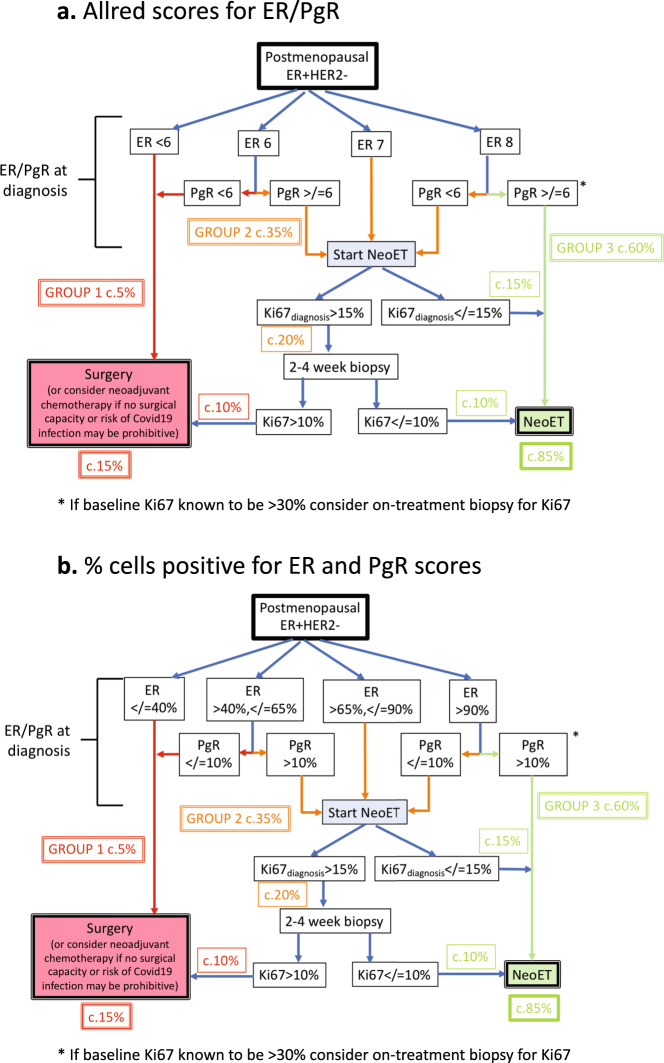
Flowcharts to show use of ER and PgR categories and on-NeoET Ki67 for directing postmenopausal patients with primary ER + HER2-breast cancer towards NeoET or surgery/neoadjuvant chemotherapy based on Allred scores or based on % cells positive. Allred scores are created by summing two separate scores: (the overall intensity of stained cells, none = 0, weak = 1, intermediate = 2, strong = 3) + (percentage of cells staining positive none = 0, <1% = 1, 1–10% = 2, 11–33% = 3, 34–66% = 4, ≥67% = 5).

The data presented in the sections below support the approach of identification of three groups of naturally postmenopausal patients that depends on the baseline expression of estrogen receptor (ER) and progesterone receptor (PgR) and an early on-treatment biopsy for Ki67 where indicated. Figure 1 summarises the approach separately for ER/PgR Allred scores (a) and percentage of cells positive (b). In some centres where Ki67 is routinely measured at diagnosis, baseline Ki67 can provide an additional means of identifying those patients who may be excluded from on-treatment biopsy.

#### Group 1: (~5%) should NOT be considered for NeoET

This is the group most likely to show no response and possible progression during protracted NeoET. Their endocrine responsiveness is poor overall, and they have a high incidence of on-treatment Ki67 > 10% which portends a poor prognosis.

#### Group 2: (~35%) may be considered for NeoET, provided that endocrine responsiveness is demonstrated as follows

In patients with Ki67 ≥ 15% at diagnosis, a core-biopsy should be taken after 2–4 weeks or later if more convenient. If on-treatment Ki67 > 10%, patients should be considered for other options such as surgery or neoadjuvant chemotherapy rather than continuing on NeoET; if on-treatment core biopsy and/or Ki67 analysis are unavailable, enhanced monitoring should be performed. Regarding the feasibility of on-treatment biopsies, if a surgical marker has not been placed, a patient can be started on NeoET and the surgical marker placed at the time of the on-treatment biopsy.

#### Group 3: (~60%) may remain on NeoET for at least 6 months

This group have very good endocrine responsiveness and a low incidence of on-treatment Ki67 > 10% overall. However, if baseline Ki67 is known to be >30%, an on-treatment biopsy should be considered as in Group 2.

Marti and Sanchez-Mendez^2^ have recently advocated the use of NeoET with biopsy for Ki67 and dichotomisation at 10% but provided no strategy for minimizing the numbers requiring on-treatment biopsy.

### ER, PgR at diagnosis and clinical response

Studies in which ER+ PgR+ or simply high-ER score tumors (eg., H-score > 100) were selected for NeoET reported <5% of tumors progressing de novo on ET^3,4^. In the first, a phase 2 study conducted in Edinburgh, all tumors were ER+ and 92% were also PgR+. Patients were treated with neoadjuvant letrozole and 23/24 (96%) had a >25% reduction in tumor volume over the 3-month study period. In a second study, only 1/47 (~2%) of tumors with an H-score of >100 had de novo progression during the preoperative phase of ET treatment. The Edinburgh group recently audited their data on 456 postmenopausal patients treated with letrozole for a mean of 206 days between 2001 and 2016. The 4% of cases with Allred ER scores of 5 or 6 showed substantially poorer regression than those with Allred scores >6 (mean absolute reduction in largest volume = 7.2% and 34.4%, respectively).

These data suggest that high initial ER levels or a combination of both ER+ and PgR+ could be used to select a group of tumors highly likely to be controlled on NeoET. This approach alone still leaves a significant proportion of ER+ tumors that may progress during NeoET, particularly if NeoET must be prolonged, and thus additional approaches to response assessment are required if NeoET is to be used optimally and without adversely affecting long-term outcomes.

### Early on-treatment Ki67 and clinical response

Presurgical use of ET can be either short-term, generally for about 2–4 weeks during the “window-of-opportunity” prior to scheduled surgery, or longer-term, usually for at least 3 months and in many cases up to 6 months and sometimes beyond^5^. The focus in the former, so-called window trials, is on obtaining biological response data including the proliferation marker Ki67. In the longer term, the primary goal of NeoET is downstaging of the disease and sometimes deriving biological response data for adjuvant treatment planning.

In several clinical trials of therapeutic NeoET, a biopsy at 2–4 weeks after starting NeoET has been taken for Ki67 measurement. There is substantial evidence for the on-treatment values providing strong prognostic information^6,7,8^. There are, however, few published data where the Ki67 data are considered in relation to clinical response or local tumor control.

We have now examined the data from two previously published NeoET clinical trials in postmenopausal patients to examine this relationship: anastrozole±gefitinib (IL1839/223)^9^ and letrozole±palbociclib (PALLET)^10^. Recent studies have consistently assessed a Ki67 cut-off at 10% as either a primary end-point or key factor in treatment allocation (POETIC^5^, WSG-ADAPT^11^, Z1031^7^, ALTERNATE^12^), and this pre-specified cut-off has been employed in all of the data analyses performed below.

Patients with HER2+ disease were excluded at recruitment from PALLET, and those entered into IL1839/223 were excluded from the current analysis. Gefitinib has no significant impact on either Ki67 or clinical response^9^, so all patients in IL1839/223 starting on anastrozole for 2 weeks were included in this analysis; for PALLET only those patients in the letrozole alone arm were included, because palbociclib has profound antiproliferative effects in addition to those seen with letrozole^10^. For each study, 4 categories of residual, tumor size at the end of NeoET were prospectively developed based on 1-dimensional ultrasound measurements: >90%; <90%, ≥70%; <70%, ≥50%; <50% of baseline. The proportion of cases with 2-week Ki67 values dichotomized at 10% in each of these categories is are shown in Table 2. It is clear that the better the Ki67 suppression at 2 weeks the greater the tumor size reduction from baseline, with the two sets of data producing similar results: in both trials, about 5 times as many patients had 2-week Ki67 > 10% among those whose tumors regressed poorly (<10% decrease), compared with those whose tumors regressed by >50%. For IL1839/223, *p*-for trend = 0.0504, and for the letrozole arm of PALLET, *p* = 0.02; summating the two sets of data, *p* = 0.002.

**Table 2:** Relationship of 2-week Ki67 and tumor shrinkage (single dimension RECIST) for IL1839/223 study, anastrozole only to 2 weeks ± gefitinib to 16 weeks, *p* for trend = 0.0504 and PALLET study (Letrozole only—group A), *p* for trend = 0.02.

Residual tum size % baseline	*n*	Ki67 > 10% *n*	Ki67 > 10% (%)
IL1839/223			
>90	22	7	32
<90, ≥70	30	7	23
<70, ≥50	37	6	16
<50	14	1	7
PALLET study			
≥90	22	8	36
<90 to ≥70	24	7	29
<70 to ≥50	20	3	15
<50	15	1	7

In the Z1031B study of neoadjuvant aromatase inhibitors^13^, of the 214 patients with Ki67 measured after 2–4 weeks, 165 (77%) patients had values ≤ 10% and of this group three patients showed progressive disease at 16 weeks. Building on these data, the ALTERNATE trial triaged postmenopausal patients with Ki67 > 10% after 4 weeks’ NeoET (anastrozole alone, fulvestrant alone, or the combination) to chemotherapy, whereas those with Ki67 ≤ 10% are maintained on anastrozole ± fulvestrant for 24 weeks before surgery. Reassuringly, only about 2% of patients maintained on NeoET experienced progression over that time, of whom half were confirmed radiologically.

Overall, these combined data provide a strong rationale for assessment of Ki67 after 2–4 weeks as a criterion for selecting patients who can safely be maintained on NeoET. However, during the COVID-19 pandemic, imaging-guided biopsies are not always readily available. In view of the direct relationship with clinical response as described above, we have evaluated whether ER and PgR expression at diagnosis can be used to reduce the number of patients for whom on-treatment biopsy is needed, and whether baseline Ki67 (if available) can further inform the decision.

### Hormone receptor status and early on-treatment Ki67: a means of triaging patients

Higher levels of ER expression are known to relate to greater proportional benefit from adjuvant endocrine therapy^14^ and to relate to greater Ki67 suppression between baseline and 2 weeks, with aromatase inhibitors or tamoxifen^15^. Although PgR+ and PgR− cases show similar proportional benefit from adjuvant tamoxifen, PgR+ tumors have better prognosis, demonstrate greater clinical response to endocrine therapy and show greater Ki67 suppression with aromatase inhibitors or tamoxifen. We have therefore examined the degree to which ER and PgR status are associated with 2-week Ki67 in POETIC, ADAPT, PALLET and IL1839/223.

Our training analysis was conducted on data from a case-control (relapses vs non-relapses) study underway in POETIC. Receptor expression categories were derived from this exercise, then subsequently tested in the other trials to examine their validity. In each case, the data were categorised according to the Allred scores and subsequently according to percent ER+ cells to provide wide applicability. For POETIC, PALLET, and IL1839/223, the Allred scores were derived as close approximations from the ER and PgR H-scores.

Table 3 shows the number and proportion of Ki67 values ≤ 10% and >10% in POETIC, according to Allred scores for ER levels and PgR, with the latter dichotomised at ≤5 vs ≥6. This dichotomy equates closely to 10% of cells PgR positive. Below, the PgR groups are termed PgR− and PgR+, according to this cut-off.

**Table 3:** Numbers (percentage) of POETIC patients with on-treatment Ki67 values >10% or ≤10%, according to ER and PgR H-scores and approximate Allred scores.

	PgR Allred < 6			PgR Allred ≥ 6			Total		
ER H-scores	Ki67 > 10%	Ki67 ≤ 10%	Total numbers	Ki67 > 10%	Ki67 ≤ 10%	Total numbers	Ki67 > 10%	Ki67 ≤ 10%	Total numbers
ER > 1 and ≤75 (Allred equiv 2–5)	15 (83%)	3 (17%)	18	1 (20%)	4 (80%)	5	16 (70%)	7 (30%)	23
ER > 75 and ≤150 (Allred equiv. 6)	7 (47%)	8 (53%)	15	5 (26%)	14 (74%)	19	12 (35%)	22 (65%)	34
ER > 150 and ≤175 (Allred equiv. 7,8)	3 (30%)	7 (70%)	10	7 (28%)	18 (72%)	25	10 (29%)	25 (71%)	35
ER > 175 and ≤200	6 (30%)	14 (70%)	20	11 (17%)	53 (83%)	64	17 (20%)	67 (80%)	84
ER > 200 and ≤225	5 (29%)	12 (71%)	17	9 (16%)	48 (84%)	57	14 (19%)	60 (81%)	74
ER > 225 and ≤250	4 (44%)	5 (56%)	9	4 (15%)	23 (85%)	27	8 (22%)	28 (78%)	36
ER > 250 and ≤275	0 (0%)	4 (100%)	4	2 (12%)	15 (88%)	17	2 (10%)	19 (90%)	21
ER > 275 and ≤300	1 (17%)	5 (83%)	6	2 (8%)	23 (92%)	25	3 (10%)	28 (90%)	31
Total ER > 175 and ≤300 (Allred equiv 8)	16 (29%)	40 (71%)	56	28 (15%)	162 (85%)	190	44 (18%)	202 (82%)	246

Cases with ER scores of ≤5 had a 65% incidence of Ki67 > 10% at 2 weeks. Tumors with an ER approximating to an Allred score of 6 but that were additionally PgR−, also had a high (47%) incidence of 2-week Ki67 > 10%. In contrast, tumors with an ER H-score of >175, approximating to an Allred score of 8, and that were also PgR+ had only a 15% incidence of 2-week Ki67 > 10%. In between these two extremes, the groups that formed Allred ER 7 or 8 but PgR− showed a 32% incidence of 2-week Ki67 > 10%, similar to the 27% incidence in those with Allred ER 6 or 7 and PgR+.

From POETIC, we have therefore defined three groups with Allred scores as shown in Table 4. Data that emerge from POETIC using these categories are shown in Table 5. Given that many centres use percentage (%age) ER/PgR scoring, we also developed groupings based on %ages based on data from POETIC shown in Table 6. These groupings are also shown in Table 4 and data that emerge from POETIC using these categories are shown in Table 5.

**Table 4:** Patient groups based on Allred scores and %age cells positive for ER and PgR.

	Allred score	%age cells positive
Group 1	ER < 6	ER ≤ 40%
	ER 6 and PgR <6	ER > 40%, ≤65% and PgR ≤ 10%
Group 2 (a)	ER7 or 8 and PgR <6	ER > 65% and PgR ≤ 10%
Group 2 (b)	ER 6 or 7 and PgR ≥ 6	ER > 40%, ≤90% and PgR > 10%
Group 3	ER 8 and PgR ≥ 6	ER > 90% and PgR > 10%

**Table 5:** Summary of groups 1, 2 and 3 for POETIC.

	Based on Allred scores			Based on % cells positive		
ER/PgR group	Ki67 > 10%	Ki67 ≤ 10%	Total	Ki67 > 10%	Ki67 ≤ 10%	Total
	*n* (%)	*n* (%)	*n*	*n* (%)	*n* (%)	*n*
1	23 (61)	15 (39)	38	17 (65)	9 (35)	26
2 (a)	19 (29)	47 (71)	66	30 (33)	62 (67)	92
2 (b)	12 (27)	32 (73)	44	11 (22)	38 (67)	49
2 (a + b)	31 (28)	79 (72)	110	41 (30)	100 (72)	141
3	28 (15)	162 (85)	190	24 (14)	148 (86)	172
Total			338			339
Remove from NeoET			69 (20%)			67 (20%)
Remain on NeoET			269 (80%)			272 (80%)

ER/PgR groupings based on Allred scores or percentage cells positive.

Values for Ki67 are on treatment.

**Table 6:** Numbers (percentage) of POETIC patients with on-treatment Ki67 values >10% or ≤10%, according to ER and PgR percentage scores and approximate Allred scores.

ER H-scores	PgR ≤ 10%			PgR >10%			Total		
	Ki67 > 10%	Ki67 ≤ 10%	Total numbers	Ki67 > 10%	Ki67 ≤ 10%	Total numbers	Ki67 > 10%	Ki67 ≤ 10%	Total numbers
*Allred equiv c.2–5*									
ER ≤ 40%	9 (75%)	3 (25%)	12	1 (25%)	3 (75%)	4	10 (63%)	6 (37%)	16
*Allred equiv c.6*									
ER > 40, ≤65%	7 (70%)	3 (30%)	10	1 (17%)	5 (83%)	6	8 (50%)	8 (50%)	16
*Allred equiv c.7*									
ER > 65, ≤90%	14 (45%)	17 (55%)	31	10 (33%)	33 (77%)	43	24 (32%)	50 (68%)	74
*Allred equiv c.8*									
ER > 90%	16 (26%)	45 (74%)	61	24 (14%)	148 (86%)	172	40 (17%)	193 (83%)	233

It can be seen in Table 5 that the proportions of patients in the groupings were very similar. We therefore tested the validity of the Allred subgroupings in the combined data from PALLET + IL1839/223 and from the ADAPT trial. For the latter, we treated the data from postmenopausal AI-treated patients separately from that of premenopausal patients, who mostly received tamoxifen (data shown in detail in Table 7 according to Allred scores). Table 8 show the data from PALLET + IL1839/223 and ADAPT postmenopausal patients, respectively. The proportion of patients with Ki67 > 10% or ≤10% in groups 1, 2, and 3 are very similar to those in POETIC. Of particular note, the proportions of patients with Ki67 > 10% in group 2 were 30% in POETIC, 32% in PALLET + IL1839/223 and 28% in ADAPT postmenopausal. In addition to percentages, the ADAPT trial also provided centrally performed Allred scores; the similarity between the study results supports the approximations to Allred scores made in POETIC, PALLET and IL1839/223.

**Table 7:** Numbers (percentage) of ADAPT patients with on-treatment Ki67 values >10% or ≤10%, according to ER and PgR Allred scores.

	PgR < 6			PgR ≥ 6			Total		
ER	Ki67 > 10%	Ki67 ≤ 10%	Total numbers	Ki67 > 10%	Ki67 ≤ 10%	Total numbers	Ki67 > 10%	Ki67 ≤ 10%	Total numbers
ADAPT postmenopausal									
Allred <6	33 (70)	14 (30)	47	5 (71)	2 (29)	7	38 (70)	16 (30)	54
Allred 6	6 (60)	4 (40)	10	7 (28)	18 (72)	25	13 (37)	22 (63)	35
Allred 7	35 (38)	58 (62)	93	52 (24)	168 (76)	220	87 (28)	226 (72)	313
Allred 8	124 (27)	331 (73)	455	184 (17)	884 (83)	1068	308 (20)	1215 (80)	1523
ADAPT premenopausal									
Allred <6	24 (75)	8 (25)	32	16 (67)	8 (33)	24	40 (71)	16 (29)	56
Allred 6	12 (75)	4 (25)	16	39 (77)	12 (23)	51	51 (76)	16 (24)	67
Allred 7	51 (73)	19 (27)	70	257 (60)	170 (40)	427	308 (62)	189 (38)	497
Allred 8	68 (68)	32 (32)	100	366 (55)	300 (45)	666	434 (57)	332 (43)	766

**Table 8:** Summary of groups 1, 2, and 3 for PALLET/IL1839/223, and ADAPT postmenopausal and premenopausal.

	PALLET/IL1839/223			ADAPT postmenopausal based on Allred scores			ADAPT premenopausal based on Allred scores		
ER/PgR group	Ki67 > 10%	Ki67 ≤ 10%	Total	Ki67 > 10%	Ki67 ≤ 10%	Total	Ki67 > 10%	Ki67 ≤ 10%	Total
	*n* (%)	*n* (%)	*n*	*n* (%)	*n* (%)	*n*	*n* (%)	*n* (%)	*n*
1	4 (44)	5 (56)	9	44 (69)	20 (31)	64	52 (72)	20 (28)	72
2 (a)	17 (32)	36 (68)	53	159 (29)	389 (71)	548	119 (70)	51 (30)	170
2 (b)	10 (31)	22 (69)	32	59 (24)	186 (76)	245	296 (62)	182 (38)	478
2 (a + b)	27 (32)	58 (68)	85	218 (28)	575 (72)	793	415 (64)	233 (36)	648
3	24 (15)	139 (85)	163	184 (17)	884 (83)	1068	366 (55)	300 (45)	666
Total			257			1925			1386
Remove from NeoET			36 (14%)			282 (15%)			–
Remain on NeoET			221 (86%)			1643 (85%)			–

ER/PgR groupings based on Allred scores. Values for Ki67 are on treatment.

Tables 5 and 8 identify the patients who, on the basis of ER/PgR baseline status and on-treatment Ki67, should be considered for longer-term NeoET or conversely should not continue on NeoET. In PALLET + IL1839/223 and ADAPT postmenopausal patients, the proportions of patients who can remain on NeoET are very similar at 86% and 85%, respectively. From the POETIC data, the proportion was a little lower, which was expected, as this is a case-controlled cohort with 50% of the population experiencing a relapse. For POETIC, the overall proportion of patients recommended for on-treatment biopsy was 33% (110/338) if based on Allred scores and 42% if based on %age of cells staining; for PALLET/ IL1839/223 the proportion was 33% (85/257), and for ADAPT postmenopausal patients it was 41% (793/1925).

### Use of baseline Ki67 for decreasing the proportion recommended for on-treatment biopsy

Baseline (i.e., pre-NeoET) Ki67 values are rarely below values obtained after short-term NeoET: in POETIC, 743/776 tumors had Ki67 ≤ 10% after 2 weeks among those that were ≤10% at diagnosis. To determine whether assessing (or when already analysed accessing) pre-NeoET Ki67 values could reduce the proportion of patients recommended for biopsy, we examined the relationship of pre-NeoET Ki67 values with on-treatment Ki67 specifically in Group 2. We applied cut-off values of 10%, 15%, and 20% to the pre-NeoET Ki67 values for three trial populations (Table 9).

**Table 9:** Relationship in Group 2 (or for Z1031B with ER Allred scores 6–8) between baseline cut-offs for Ki67 and the proportion of patients with Ki67 ≤ 10% or >10%.

	POETIC (Group 2 by Allred scores)			Z1031B			ADAPT postmenopausal (Group 2 by percentage +ve cells)		
Pre-NET Ki67	Group 2 split	week-2 Ki67		Group 2 split	week-3 Ki67		Group 2 split	week-4 Ki67	
Cut-off	*n* (% of group 2)	*n* (% of group split)		*n* (% of group 2)	*n* (% of group split)		*n* (% of group 2)	*n* (% of group split)	
		≤10%	>10%		≤10%	>10%		≤0%	>10%
≤10%	25 (23%)	25 (100%)	0 (0%)	68 (33%)	60 (88%)	8 (12%)	251 (27.3%)	230 (91.6%)	21 (8.4%)
>10%	85 (77%)	54 (64%)	31 (36%)	139 (67%)	101 (73%)	38 (27%)	668 (72.3%)	434 (65.0%)	234 (35.0%)
≤15%	43 (39%)	39 (91%)	4 (9%)	113 (55%)	100 (88%)	13 (12%)	439 (47.8%)	381 (86.8%)	58 (13.2%)
>15%	67 (61%)	40 (60%)	27 (40%)	94 (45%)	61 (65%)	33 (35%)	480 (52.2%)	283 (59.0%)	197 (41.0%)
≤20%	58 (53%)	50 (86%)	8 (14%)	136 (66%)	121 (89%)	15 (11%)	594 (64.6%)	491 (82.7%)	103 (17.3%)
>20%	52 (47%)	29 (56%)	23 (44%)	71 (34%)	40 (56%)	31 (44%)	325 (35.4%)	173 (53.2%)	152 (46.8%)
No-cut-off	110 (100%)	79 (72%)	31 (28%)	207 (100%)	161 (73%)	46 (27%)	919 (100%)	664 (72.3%)	255 (27.7%)

The bottom line of each panel shows the proportion of patients that would have on-treatment Ki67 in these categories in the absence of using a baseline Ki67 cut-off.

In POETIC, 43 (39%) of the Group 2 population had on-treatment Ki67 values ≤ 10% if they had values ≤ 15% at diagnosis. Four (9%) of the 43 had values above 10% at 2 weeks. In contrast, in the 27/67 (40%) patients with pre-NeoET Ki67 values >15% had on-NeoET Ki67 values of ≤10%.

In ADAPT post-menopausal patients, only 44/566 (7.8%) with baseline Ki67 ≤ 10% had an on-treatment Ki67 > 10%. Patients in Group 2 with baseline Ki67 ≤ 15% had a > 85% likelihood of obtaining on-treatment Ki67 ≤ 10%. This represent 44% of Group 2 patients (of note, the cutoff of 15% also applies if ER and PgR measurements were expressed according to Allred score).

Similar proportions of patients can be seen in each of the subdivisions of the Z1031B population with ER Allred scores of 6–8.

With regard to Group 3, post-menopausal ADAPT patients with baseline Ki67 measurements ≤ 30% had a > 85% likelihood of obtaining on-treatment Ki67 ≤ 10%. However, among patients with baseline Ki67 > 30% (who comprise only ~20% of Group 3), only about 57% obtained on-treatment Ki67 ≤ 10%. Thus, for this subset of Group 3, the authors recommend obtaining an on-treatment Ki67 measurement (if available) to ensure endocrine sensitivity before continuing NeoET.

### Premenopausal patients

The safety concerns from COVID-19 infection are less in younger women and there are far fewer data on the long-term clinical efficacy of NeoET in premenopausal women. There are also fewer data on the short-term effects on Ki67 than in postmenopausal women. Consistent suppression of Ki67 was seen with both tamoxifen and a single 750 mg dose of fulvestrant in an Edinburgh study with tamoxifen showing a trend for greater suppression^16^. DeCensi reported that Ki67 was suppressed by tamoxifen after 4 weeks in premenopausal women, but the values were higher than those in postmenopausal women both before and at the end of treatment^8^. These findings are consistent with the higher proportion (60.5% vs. 24.4%) of pre- (88.6% treated with tamoxifen) vs. postmenopausal patients (91.9% treated with AI) in ADAPT having on-treatment Ki67 > 10%. The difference may result from Ki67 not being fully suppressed by tamoxifen within 3 weeks, due to the much higher competing estrogen levels in premenopausal women.

In Table 8, it can be seen that there were no strong relationships between on-treatment Ki67 and ER or PgR levels in premenopausal patients in ADAPT, so these levels therefore cannot be recommended to reduce the number of patients who could be spared an on-treatment biopsy, even considering baseline Ki67.

Overall, it seems reasonable to use on-treatment Ki67 if there is a clinical need to prioritise NeoET for premenopausal patients, but there are few clinical data to directly support this so we are unable to recommend this. In ADAPT pre-menopausal patients, the percentages of patients with on-treatment Ki67 ≤ 10% were 25.8%, 37.0%, and 43.6% in Groups 1, 2, and 3, respectively.

### Enhanced monitoring when on-treatment biopsy for Ki67 is unavailable

During the COVID-19 pandemic, resources for imaging-guided biopsies may be restricted and on-treatment biopsy for Ki67 unavailable for some patients. In the Edinburgh group’s audit of 456 postmenopausal patients treated with letrozole, a reduction of at least 15% in relative tumor volume early in treatment (mean = day 47) was significantly associated with continued neoadjuvant clinical response. Thus, for those patients in whom an on-treatment biopsy would otherwise be assessed, an interval assessment of maximum tumor size as measured clinically and by ultrasound or mammogram could be considered as an alternative. Failure to reduce maximum volume by ≥15% at 6 weeks and ≥20% at 3 months is associated with a significantly lower chance of long-term local control and a worse long-term survival. An on-treatment biopsy is preferred if resources and safety allow this.

### Reliability of Ki67 analysis

It has been widely reported that Ki67 results can vary substantially between centres^17^. However, in an early study by the International Ki67 in Breast Cancer Working Group (KiBCWG)^18^, analysts at expert labs were found to have excellent within-lab consistency (ICC 0.94). Although the labs involved in the studies considered in the current report have not undertaken formal exchange of materials, there is evidence for their measurements being similar: the data relationships between steroid receptor subgroups and on-treatment Ki67 is very similar; and the ADAPT run-in phase almost exactly reproduced Ki67 assumptions based on the data from the Ellis and Dowsett labs^19^. Moreover, the analyses reported above for the ADAPT trial provided similar results using locally vs. centrally determined ER, PgR, and baseline Ki67.

Centres wishing to apply on-treatment Ki67 should confirm that their Ki67 analysis is operating to similar levels and should contact the authorship to access material if needed to do so. Differences in scoring of Ki67 were found by the International KiBCWG to be the most important source of between-laboratory variability. Consistency of scores is markedly enhanced by adherence to the methodology for “global scoring” which was validated and endorsed by the KiBCWG^20^ and is advocated here. Using this method, the median baseline Ki67 in ER + HER2- tumors in the whole POETIC trial (ie., not just the case-control series described here) was 14.3% (*n* = 3452). In ADAPT, median baseline Ki67 was 20% for both pre- and postmenopausal patients (*n* = 1954 and 2730, respectively). The median baseline Ki67 in ADAPT was expected to be higher than in POETIC since eligibility for ADAPT was confined to those that were candidates for (neo)adjuvant chemotherapy by conventional prognostic criteria.

### Clinical factors

Clinical factors such as tumor size and nodal status do not impact on response to NeoET but do relate to the overall prognosis of patients. Their interaction with competing factors of advanced age/frailty and co-morbidity is helpful for decision-making in individual patients, and assists clinicians in prioritising the individual for surgery within a larger cohort of patients competing for appropriate care as in the COVID-19 pandemic. Thus, while the clinician should take these features into account when making decisions on appropriateness of NeoET the complexity of the interactive factors precludes our making precise recommendations.

The appropriateness or advantage of continued NeoET should be individualised and needs to re-appraised with frequent regularity as the situation changes in times of a pandemic such as the current one. While we do not recommend fixed durations for treatment, if the objective is to promote breast conserving surgery, 4–6 months is typical and longer can be helpful for larger tumors. In patients with smaller tumors who are already candidates for breast conservation, surgery could be as soon as the availability of resources returns.

The preoperative endocrine prognostic index (PEPI) was derived from patients in clinical trials where the length of NeoET varied between 12 and 18 weeks (12 weeks IMPACT/P024 and 16–18 weeks for Z1031^7^). If a longer or shorter period of NeoET is used, the PEPI approach could still be applied on an individual basis, since the Ki67 is prognostic after very short periods of time (2 to 4 weeks). Thus, if a patient’s surgical sample was assigned PEPI-0 (Pathological stage T1 or T2, N0, Ki67 < 2.7%) and surgery was conducted when the patients was still on NeoET, post-surgical management without chemotherapy could be considered.

### Genomic assays and use of NeoET

Genomic assays are in widespread use for the selection of those patients with ER+ HER2− node-negative breast cancer who should receive adjuvant chemotherapy as well as endocrine therapy. They estimate risk of distant recurrence on endocrine therapy alone largely by integrating information on baseline proliferation and features relating to the likelihood of endocrine responsiveness. Their main objective is to aid in counselling patients regarding their individual risk/benefit ratio for the use of chemotherapy. In contrast to the data presented here, there are no data on the relationship of genomic assays and local control with NeoET. Our proposal also has the advantage of undertaking an on-treatment measurement of the response to endocrine therapy (2–4 week Ki67). If a genomic assay is undertaken it must be conducted on a biopsy before starting NeoET since this has a profound but so far ill-described effect on the expression of many genes in the tests.

## Conclusions

In summary, based on tumor ER and/or PgR expression at diagnosis and (if available) baseline Ki67, postmenopausal patients with hormone receptor positive HER2-negative early breast cancer can be stratified for immediate surgery or neoadjuvant chemotherapy (Group 1) and others selected for NeoET (Group 3). Management of the remaining patients (Group2) is dependent on Ki67 assessment. Group 2 patients with baseline Ki67 ≤ 15% may continue on NeoET, while the remainder should be considered for a biopsy for Ki67 at 2–4 weeks to see if Ki67 ≤ 10%, so they can continue on NeoET. Group 2 patients whose on-treatment Ki67 is >10% should not continue on NeoET.

NeoET may also be an option for premenopausal patients and on-treatment Ki67 may be a helpful guide.

We propose the flow diagrams based on these groupings (Fig. 1a, b) to assist clinicians in the selection of postmenopausal patients likely to have acceptable outcomes on NeoET and at the same time prioritise a smaller group for surgery if operating room capacity allows, or for neoadjuvant chemotherapy as an alternative.

The data underpinning these recommendations are strong for postmenopausal but more limited for premenopausal patients. This guidance needs to be interpreted in the light of other clinical features. We have already emphasised that at present these recommendations are designed to help manage breast cancer only during the current COVID-19 crisis or during further waves. It might of course in time prove to have a role in the standard management of some patients with early breast cancer; this will require validation by clinical trials.

## Methods

### Data sets analysed

The authors identified sets of their NeoET trial data both published and unpublished on which ER, PgR and Ki67 at diagnosis and Ki67 after 2–4 weeks’ NeoET were available. Only clinical response data were available from the on-going ALTERNATE trial. The PALLET and IL1839/223 trials had clinical response data as well as immunohistochemical data.

The clinical trials are listed in Table 1 along with key design issues and the number of patients that were included in the current paper from each. The citations are given for each trial where the details of design and inclusion/exclusion criteria are given along with the primary outcome data for those trials that have completed and reported.

The data on POETIC were from a case-control study in which cases were patients with recurrences within the first 5 years of follow-up with a case:control ratio of 1:1.

We also accessed data from an audit of 454 patients treated in Edinburgh between 2001 and 2016 with neoadjuvant letrozole for a mean 206 days.

All relevant ethical regulations were complied with and the name of the board and institution that approved the study protocol, is included in Table 1. The POETIC trial was approved by the London-South East Research Ethics Committee. All patients gave informed consent within each of the trials for the derivation of the data sourced for this study.

### Immunohistochemical scoring

All of the immunohistochemical analyses were conducted centrally for the respective trials. Ki67 scores always given as %age of cells positive. In POETIC and PALLET ER was scored as H-score with the scores which range from 0 to 300 being the product of the %age of cells staining at given staining intensity as follows: 1 × %age of cells staining weakly + 2 × %age of cells staining moderately + 3 × %age of cells staining strongly. PgR was scored as %age of cells staining positive. In IL1839/223 both ER and PgR were scored as H-scores. In ADAPT ER and PgR were scored as Allred scores. In Z1031B ER was scored by Allred score; PgR was not analysed. For trials where the H-score was calculated the %age of cells staining (at any intensity) is also available.

### Data analyses

Some of the data included have been previously published but subgroupings and bespoke analyses were conducted specifically for this report and have not been shown previously. Data were only included in this report if all results for each of the biomarkers were available for a given patient.

The data from the POETIC case-control study acted as the training set for setting cut-offs for ER and PgR for achieving acceptable rates of Ki67 ≤ 10%. ER H-score groupings of >1 and < /=75, >75 and < /150, >150 and ≤ 175, >175 and ≤200, >200, and ≤225, >225 and ≤250, >250 and ≤275, and >275 were created. Approximate Allred equivalents were calculated as Allred 2–5 (H-score >1 and ≤75), Allred 6 (H-score >75 and ≤150), Allred 7 and 8 (H-score >175). Examination of the rates of Ki67 > 10% led to the definition of Groups 1, 2, and 3. These Allred groupings were then tested in the ADAPT, Z1031B and PALLET and IL1839/223 data combined to increase the numbers available.

The analysis of the relationship of 2 week Ki67 values and clinical response was conducted in the PALLET and IL1839/223 trials having prespecified residual tumor size cut-offs of >90%, <90% and ≥70%, <70% and ≥50%, and >50% according to one-dimensional ultrasound measurements.

All data are descriptive. The baseline Ki67 levels of the POETIC and ADAPT trials were provided as medians.

### Reporting summary

Further information on research design is available in the Nature Research Reporting Summary linked to this article.

## Supplementary information

Reporting Summary Form

## Data Availability

The data generated and analysed during this study are described in the following metadata record: 10.6084/m9.figshare.12287699 ^21^. The majority of the datasets supporting the findings of this study are derived from clinical trials that have not yet been published. Other datasets are yet to be published in association with trial secondary endpoints. For these reasons, the data supporting the findings of this study are not publicly available, but will be made available on reasonable request from the corresponding author, Professor Mitch Dowsett, email: mitchell.dowsett@icr.ac.uk. Datasets that relate to the Z1031 clinical trial will be made available to researchers on reasonable request, by contacting the ALLIANCE Operations office (email: concepts@allianceNCTN.org) and completing an Alliance Data Sharing Request Form.
